# Supplementary material for: Co-Pyrolysis of Unsaturated C4 and Saturated C6+ Hydrocarbons—An Experimental Study to Evaluate Steam-Cracking Performance
Source: Materials (Basel). 2023 Feb 8;16(4):1418. doi: 10.3390/ma16041418 (PMC9962995; doi:10.3390/ma16041418)
Supplement: Supplementary file 1 [file materials-16-01418-s001.zip › materials-2177521-supplementary.pdf]

Table S1

Co-pyrolysis of 1-butene and cyclohexane at 820 °C. Composition of reaction mixture after co-pyrolysis (in columns – content of 1-butene in the feedstock)

1-butene, %	3.9	16.1	19.5	25.9	63.2	77.9
**Hydrogen**	1.0	0.7	0.7	0.6	0.2	0.1
**Methane**	3.5	6.1	6.5	7.4	11.3	12.3
**Ethane**	0.5	0.7	0.7	0.8	1.4	1.6
**Ethylene**	31.0	33.8	33.2	33.2	30.1	28.2
**Propane**	tr.	0.1	0.1	0.1	0.1	0.1
**Propylene**	5.6	8.3	9.0	10.1	15.1	16.9
**Acethylene**	1.1	1.2	1.2	1.3	1.5	1.5
**Isobutane**	tr.	tr.	tr.	tr.	tr.	tr.
**Propadiene**	0.2	0.2	0.3	0.3	0.5	0.6
**n-Butane**	tr.	tr.	tr.	tr.	0.1	0.1
***(E)*-2-Butene**	0.1	0.2	0.2	0.3	0.5	0.5
**1-Butene**	0.4	0.6	0.7	0.8	1.6	1.9
**Isobutene**	tr.	0.1	0.1	0.1	0.2	0.2
***(Z)*-2-Butene**	0.1	0.2	0.2	0.2	0.4	0.5
**Propyne**	0.4	0.5	0.5	0.5	0.8	0.9
**Butadiene**	22.9	21.3	21.1	20.2	17.5	16.6
**Cyclohexane**	24.0	14.7	13.9	11.5	3.7	2.1
**Cyclopentadiene**	1.4	1.6	1.6	1.7	1.7	2.0
**Other NA C5–C6**	2.0	2.0	2.1	2.3	2.1	2.2
**Benzene**	4.4	5.7	5.7	6.2	7.3	7.9
**Toluene**	0.5	0.7	0.7	0.8	0.6	1.4
**Ethylbenzene**	tr.	tr.	tr.	tr.	0.1	0.1
**m- +p-Xylene**	tr.	tr.	tr.	tr.	0.1	0.1
**Styrene+o-Xylene**	tr.	tr.	tr.	tr.	0.5	0.6
**Naphthalene**	tr.	tr.	tr.	tr.	0.1	0.2
**Other C7–C12**	0.8	1.2	1.3	1.3	1.6	1.1
**C12+**	0.1	0.2	0.2	0.3	0.9	0.5

Table S2

Co-pyrolysis of 2-butene and hexane at 820 °C. Composition of reaction mixture after co-pyrolysis (in columns – content of 2-butene in the feedstock)

2-butene, %	4.2	15.2	19.2	23.7	27.5	62.4	82.2	100.0
**Hydrogen**	0.8	0.6	0.5	0.3	0.1	0.3	0.2	0.3
**Methane**	12.3	13.2	13.4	14.3	15.5	12.3	12.2	9.3
**Ethane**	2.5	2.5	2.5	2.6	2.6	1.7	1.1	0.7
**Ethylene**	56.6	54.1	53.3	53.4	52.1	30.6	19.8	7.2
**Propane**	0.4	0.3	0.3	0.3	0.2	0.1	0.1	tr.
**Propylene**	13.7	14.1	14.1	13.8	13.7	18.5	18.9	18.2
**Acethylene**	0.8	1.1	1.2	1.5	1.6	1.2	1.3	1.1
**Isobutane**	0.3	0.3	0.3	0.3	0.3	0.4	0.4	0.5
**Propadiene**	tr.	tr.	tr.	tr.	tr.	tr.	tr.	tr.
**n-Butane**	tr.	tr.	tr.	tr.	tr.	tr.	tr.	tr.
***(E)*-2-Butene**	0.2	0.4	0.4	0.4	0.4	3.1	5.0	11.8
**1-Butene**	1.5	1.4	1.4	1.4	1.4	2.3	2.2	2.9
**Isobutene**	0.1	0.1	0.1	0.1	0.1	0.2	0.3	0.4
***(Z)*-2-Butene**	0.2	0.3	0.4	0.4	0.4	2.7	4.3	10.1
**Propyne**	0.3	0.5	0.5	0.6	0.6	0.7	0.9	1.0
**Butadiene**	4.6	6.0	6.4	6.3	6.5	16.2	21.5	27.3
**Hexane**	3.0	2.0	1.9	1.5	1.1	2.0	0.9	tr.
**Cyclopentadiene**	0.3	0.3	0.3	0.2	0.2	1.2	1.5	1.2
**Other NA C5–C6**	0.6	0.7	0.7	0.6	0.7	1.9	2.7	2.9
**Benzene**	1.1	1.3	1.4	1.4	1.5	2.9	4.0	2.5
**Toluene**	0.2	0.2	0.3	0.2	0.2	0.6	0.9	0.6
**Ethylbenzene**	tr.	tr.	tr.	tr.	tr.	0.1	0.1	0.1
**m- +p-Xylene**	tr.	tr.	tr.	tr.	tr.	0.1	0.1	0.1
**Styrene+o-Xylene**	tr.	0.1	0.1	0.1	0.1	0.2	0.3	0.3
**Naphthalene**	tr.	tr.	tr.	tr.	tr.	tr.	tr.	tr.
**Other C7–C12**	0.2	0.3	0.3	0.2	0.2	0.6	0.8	1.1
**C12+**	0.2	0.2	0.2	0.2	0.6	0.2	0.3	0.5

Table S3

Co-pyrolysis of 2-butene and cyclohexane at 820 °C. Composition of reaction mixture after co-pyrolysis (in columns – content of 2-butene in the feedstock)

2-butene, %	10.6	14.2	17.9	21.8	27.6	30.5	75.3
**Hydrogen**	0.9	0.8	0.8	0.7	0.6	0.7	0.4
**Methane**	4.3	4.6	4.5	5.4	6.4	6.7	8.7
**Ethane**	0.4	0.4	0.3	0.4	0.4	0.5	0.5
**Ethylene**	29.2	28.2	25.7	25.6	26.8	26.0	13.0
**Propane**	tr.	tr.	tr.	0.1	tr.	0.1	tr.
**Propylene**	7.0	7.6	7.9	9.3	10.0	10.6	16.4
**Acethylene**	1.1	1.1	1.0	1.1	1.0	1.1	1.1
**Isobutane**	tr.	tr.	tr.	tr.	tr.	tr.	0.4
**Propadiene**	0.2	0.2	0.2	0.3	0.2	0.2	tr.
**n-Butane**	tr.	tr.	tr.	tr.	tr.	tr.	tr.
***(E)*-2-Butene**	0.8	1.1	1.5	1.9	2.0	2.1	7.9
**1-Butene**	0.4	0.5	0.6	0.7	0.7	0.7	2.2
**Isobutene**	0.1	0.1	0.1	0.1	0.1	0.1	0.3
***(Z)*-2-Buten**	0.7	0.9	1.2	1.6	1.6	1.7	6.8
**Propyne**	0.4	0.4	0.4	0.5	0.4	0.5	0.8
**Butadiene**	23.4	23.2	22.8	23.4	22.3	23.3	25.6
**Cyclohexane**	22.0	22.4	25.1	20.2	19.0	14.7	6.3
**Cyclopentadiene**	1.1	1.1	0.9	1.0	0.9	1.2	1.5
**Other NA C5–C6**	1.8	1.8	1.8	1.9	1.8	2.1	2.9
**Benzene**	4.4	4.1	3.7	4.0	4.2	4.9	3.3
**Toluene**	0.5	0.4	0.4	0.5	0.5	0.7	0.7
**Ethylbenzene**	tr.	tr.	tr.	tr.	tr.	0.1	0.1
**m- +p-Xylene**	0.1	tr.	tr.	0.1	0.1	0.1	0.1
**Styrene+o-Xylene**	0.3	0.2	0.2	0.2	0.2	0.3	0.2
**Naphthalene**	0.1	tr.	tr.	0.1	tr.	0.1	tr.
**Other C7–C12**	0.6	0.6	0.6	0.6	0.6	0.9	0.7
**C12+**	0.3	0.3	0.3	0.3	0.3	0.4	0.2

Table S4

Co-pyrolysis of isobutene and hexane at 820 °C. Composition of reaction mixture after co-pyrolysis (in columns – content of isobutene in the feedstock)

Isobutene, %	4.6	9.6	14.4	20.6	22.3	43.0	55.8	73.4	100.0.
**Hydrogen**	0.9	0.8	0.8	0.5	0.6	0.3	0.2	tr.	tr.
**Methane**	12.2	12.5	12.6	13.7	13.2	14.0	13.5	12.6	7.1
**Ethane**	2.3	2.4	2.3	2.0	2.1	1.7	1.5	1.2	0.4
**Ethylene**	55.9	53.0	51.1	48.7	47.8	37.6	29.8	19.8	0.9
**Propane**	0.3	0.3	0.3	0.3	0.3	0.2	0.1	0.1	tr.
**Propylene**	13.8	14.2	13.9	13.7	14.1	13.1	11.9	10.0	3.7
**Acethylene**	0.8	1.1	1.0	0.8	0.9	0.8	0.9	0.7	0.5
**Isobutane**	tr.	tr.	tr.	tr.	tr.	tr.	tr.	tr.	tr.
**Propadiene**	0.4	0.6	0.8	0.9	0.9	1.7	2.6	3.7	5.6
**n-Butane**	tr.	tr.	tr.	tr.	tr.	tr.	tr.	tr.	tr.
***(E)*-2-Butene**	0.2	0.2	0.2	0.2	0.2	0.2	0.2	0.2	0.9
**1-Butene**	1.6	1.7	1.6	1.4	1.6	1.4	1.3	1.1	0.6
**Isobutene**	1.1	2.3	3.5	5.2	5.2	13.0	19.7	32.0	65.6
***(Z)*-2-Butene**	0.2	0.2	0.2	0.2	0.2	0.2	0.2	0.3	1.0
**Propyne**	0.5	0.9	1.0	1.0	1.2	1.9	3.0	3.7	4.5
**Butadiene**	4.1	4.0	3.9	3.8	3.9	3.1	2.5	1.7	1.4
**Hexane**	3.0	2.6	2.6	2.2	2.5	2.0	2.0	1.9	tr.
**Cyclopentadiene**	0.4	0.5	0.6	1.0	0.9	1.5	1.7	1.7	0.8
**Other NA C5–C6**	0.6	0.7	0.8	1.0	1.0	1.6	2.1	2.5	2.4
**Benzene**	1.2	1.5	1.7	2.3	2.2	3.7	4.1	3.9	1.9
**Toluene**	0.2	0.2	0.4	0.5	0.6	1.2	1.5	1.6	0.9
**Ethylbenzene**	tr.	tr.	tr.	tr.	tr.	0.1	0.1	0.1	0.1
**m- +p-Xylene**	tr.	tr.	tr.	tr.	tr.	0.1	0.1	0.1	0.1
**Styrene+o-Xylene**	tr.	tr.	0.1	0.1	0.1	0.2	0.2	0.2	0.1
**Naphthalene**	tr.	tr.	tr.	tr.	tr.	tr.	tr.	tr.	tr.
**Other C7–C12**	tr.	tr.	0.4	0.1	0.2	0.3	0.6	0.7	1.2
**C12+**	0.1	0.3	0.2	0.1	0.2	0.2	0.3	0.2	0.4

Table S5

Co-pyrolysis of isobutene and cyclohexane at 820 °C. Composition of reaction mixture after co-pyrolysis (in columns – content of isobutene in the feedstock)

Isobutene, %	2.3	8.0	14.3	18.6	22.2	27.3	57.9	77.7
**Hydrogen**	0.9	0.8	0.7	0.6	0.6	0.5	0.2	tr.
**Methane**	1.9	2.5	2.8	3.0	3.3	3.6	5.8	7.0
**Ethane**	0.3	0.3	0.2	0.2	0.2	0.2	0.3	0.4
**Ethylene**	23.9	23.0	20.5	19.3	17.9	16.7	10.0	5.8
**Propane**	tr.	tr.	tr.	tr.	tr.	tr.	tr.	tr.
**Propylene**	4.1	4.5	4.6	4.7	4.8	4.9	5.3	4.9
**Acethylene**	0.9	0.8	0.8	0.8	0.7	0.7	0.6	0.4
**Isobutane**	tr.	tr.	tr.	tr.	tr.	tr.	tr.	tr.
**Propadiene**	0.3	0.4	0.6	0.8	0.9	1.1	2.7	3.7
**n-Butane**	tr.	tr.	tr.	tr.	tr.	tr.	tr.	tr.
***(E)*-2-Butene**	0.1	0.1	0.1	0.1	0.1	0.1	0.2	0.3
**1-Butene**	0.3	0.3	0.4	0.4	0.4	0.4	0.5	0.5
**Isobutene**	1.1	4.2	7.7	10.2	12.7	15.9	37.0	50.6
***(Z)*-2-Butene**	0.1	0.1	0.1	0.1	0.1	0.1	0.3	0.4
**Propyne**	0.2	0.4	0.6	0.7	0.8	1.0	2.2	3.1
**Butadiene**	20.2	19.8	18.3	17.3	16.5	15.6	9.7	5.6
**Cyclohexane**	40.0	37.0	37.5	36.3	35.5	33.9	19.2	10.4
**Cyclopentadiene**	0.8	0.8	0.7	0.8	0.7	0.7	0.9	1.0
**Other NA C5–C6**	1.6	1.7	1.5	1.5	1.5	1.5	1.9	2.1
**Benzene**	2.4	2.3	2.0	1.9	1.8	1.7	1.7	1.8
**Toluene**	0.3	0.3	0.3	0.3	0.3	0.3	0.6	0.8
**Ethylbenzene**	tr.	tr.	tr.	tr.	tr.	tr.	tr.	tr.
**m- +p-Xylene**	tr.	tr.	tr.	tr.	0.1	0.1	0.1	0.1
**Styrene+o-Xylene**	0.1	0.1	0.1	0.1	0.1	0.1	0.1	0.1
**Naphthalene**	tr.	tr.	tr.	tr.	tr.	tr.	tr.	tr.
**Other C7–C12**	0.6	0.5	0.4	0.6	0.7	0.5	0.6	0.8
**C12+**	0.1	0.1	0.1	0.1	0.2	0.1	0.1	0.1

Table S6

Co-pyrolysis of butane and hexane at 820 °C. Composition of reaction mixture after co-pyrolysis (in columns – content of butane in the feedstock)

Butane, %	3.8	14.3	22.9	28.8	31.7	48.1	61.8	83.3	100.0.
**Hydrogen**	0.9	0.9	0.9	1.1	1.0	1.0	1.1	1.0	1.1
**Methane**	12.8	13.7	13.6	12.4	13.4	13.9	13.6	16.1	15.4
**Ethane**	2.6	2.5	2.3	2.4	2.4	2.4	2.3	2.3	2.4
**Ethylene**	57.1	55.0	53.4	53.2	52.2	50.4	48.6	45.0	45.0
**Propane**	0.3	0.3	0.3	0.4	0.3	0.3	0.3	0.3	0.3
**Propylene**	13.6	14.0	14.8	14.7	15.1	15.5	16.0	16.9	16.5
**Acethylene**	1.2	1.0	0.7	0.9	0.8	0.8	0.7	0.6	0.7
**Isobutane**	0.3	0.3	0.2	0.3	0.3	0.3	0.2	0.2	0.2
**Propadiene**	tr.	tr.	tr.	tr.	tr.	tr.	tr.	tr.	tr.
**n-Butane**	0.5	2.0	3.0	4.3	4.3	6.3	9.2	10.2	13.5
***(E)*-2-Butene**	0.2	0.2	0.2	0.2	0.2	0.2	0.2	0.2	0.2
**1-Butene**	1.7	1.4	1.5	1.6	1.6	1.6	1.7	1.5	1.4
**Isobutene**	0.1	0.1	0.1	0.1	0.1	0.1	0.1	0.1	0.1
***(Z)*-2-Butene**	0.1	0.2	0.2	0.2	0.2	0.2	0.2	0.2	0.2
**Propyne**	0.4	0.4	0.3	0.4	0.4	0.4	0.3	0.3	0.3
**Butadiene**	3.9	3.9	3.9	3.6	3.8	3.4	2.9	2.8	2.0
**Hexane**	2.5	1.5	1.9	2.1	1.7	1.2	1.1	0.3	tr.
**Cyclopentadiene**	0.2	0.4	0.4	0.3	0.4	0.3	0.2	0.3	0.2
**Other NA C5–C6**	0.5	0.6	0.6	0.6	0.6	0.5	0.4	0.5	0.3
**Benzene**	0.8	1.0	1.0	0.8	0.9	0.7	0.5	0.7	0.3
**Toluene**	0.1	0.2	0.2	0.1	0.2	0.1	0.1	0.1	tr.
**Ethylbenzene**	tr.	tr.	tr.	tr.	tr.	tr.	tr.	tr.	tr.
**m- +p-Xylene**	tr.	tr.	tr.	tr.	tr.	tr.	tr.	tr.	tr.
**Styrene+o-Xylene**	tr.	tr.	tr.	tr.	tr.	tr.	tr.	tr.	tr.
**Naphthalene**	tr.	tr.	tr.	tr.	tr.	tr.	tr.	tr.	tr.
**Other C7–C12**	0.1	0.3	0.2	0.2	0.2	0.2	0.1	0.1	tr.
**C12+**	0.1	0.2	0.1	0.1	0.1	0.1	0.1	0.1	tr.

Table S7

Co-pyrolysis of butane and cyclohexane at 820 °C. Composition of reaction mixture after co-pyrolysis (in columns – content of butane in the feedstock)

Butane, %	0.8	7.6	15.1	22.5	26.4	29.5	41.8	56.9	71.4
**Hydrogen**	1.0	1.1	1.1	1.0	1.0	1.0	1.1	0.9	0.9
**Methane**	2.6	5.3	6.0	6.6	8.1	8.5	8.9	12.0	13.6
**Ethane**	0.4	0.8	0.8	0.9	1.0	1.0	1.1	1.5	1.7
**Ethylene**	30.8	39.0	41.6	40.6	43.8	44.8	43.1	46.1	46.0
**Propane**	tr.	0.1	0.1	0.1	0.1	0.1	0.1	0.2	0.2
**Propylene**	4.5	6.0	6.7	7.8	8.4	8.1	9.8	11.7	13.5
**Acethylene**	1.1	1.9	2.0	1.4	2.0	1.4	1.4	1.1	1.0
**Isobutane**	0.2	0.2	0.2	0.2	0.2	0.2	0.2	0.2	0.2
**Propadiene**	tr.	tr.	tr.	tr.	tr.	tr.	tr.	tr.	tr.
**n-Butane**	0.3	1.6	2.8	4.9	3.9	4.6	6.6	7.5	8.7
***(E)*-2-Butene**	0.1	0.1	0.1	0.2	0.1	0.1	0.2	0.2	0.2
**1-Butene**	0.3	0.3	0.4	0.5	0.5	0.5	0.7	0.8	1.0
**Isobutene**	tr.	tr.	tr.	tr.	0.1	tr.	0.1	0.1	0.1
***(Z)*-2-Butene**	0.1	0.1	0.1	0.1	0.1	0.1	0.1	0.1	0.2
**Propyne**	0.1	0.5	0.5	0.4	0.5	0.3	0.4	0.3	0.4
**Butadiene**	22.4	19.1	17.7	16.9	14.7	14.0	12.8	8.7	6.6
**Cyclohexane**	27.2	11.0	9.3	8.3	5.0	5.4	4.5	2.2	1.2
**Cyclopentadiene**	1.1	1.2	0.9	1.5	0.9	1.3	1.2	0.7	0.6
**Other NA C5–C6**	2.0	1.7	1.5	1.6	1.5	1.3	1.3	0.9	0.8
**Benzene**	4.1	6.6	5.5	5.2	5.6	5.7	4.7	3.6	2.6
**Toluene**	0.4	0.8	0.6	0.6	0.6	0.6	0.5	0.4	0.4
**Ethylbenzene**	tr.	0.1	0.1	tr.	0.1	tr.	tr.	tr.	tr.
**m- +p-Xylene**	0.1	0.1	0.1	0.1	0.1	0.1	0.1	tr.	tr.
**Styrene+o-Xylene**	0.2	0.4	0.3	0.3	0.4	0.3	0.2	tr.	tr.
**Naphthalene**	tr.	tr.	tr.	tr.	tr.	tr.	tr.	tr.	tr.
**Other C7–C12**	0.7	1.4	1.4	0.7	1.1	0.6	0.6	0.8	0.1
**C12+**	0.1	0.4	0.3	0.2	0.2	tr.	0.2	0.2	0.1

Table S8

Co-pyrolysis of isobutane and hexane at 820 °C. Composition of reaction mixture after co-pyrolysis (in columns – content of isobutane in the feedstock)

Isobutane, %	4.0	7.7	14.5	17.9	23.3	27.9	39.3	53.7	65.5	74.4	100.0
**Hydrogen**	0.8	0.9	1.0	0.9	1.0	0.9	1.1	1.0	1.1	1.2	0.8
**Methane**	13.4	12.6	13.0	13.3	12.9	13.8	13.4	14.7	15.3	15.5	18.7
**Ethane**	2.4	2.5	2.5	2.5	2.5	2.6	2.6	2.6	2.6	2.5	2.8
**Ethylene**	55.5	55.3	52.9	50.8	47.8	46.6	41.7	34.8	29.5	24.9	12.5
**Propane**	0.4	0.4	0.4	0.4	0.4	0.4	0.5	0.4	0.5	0.5	0.5
**Propylene**	14.3	14.1	14.8	15.3	16.2	16.4	17.9	19.7	21.1	22.1	25.3
**Acethylene**	0.8	1.2	1.2	0.8	0.8	0.8	1.0	0.9	1.0	1.0	0.6
**Isobutane**	0.5	1.1	1.8	2.5	3.7	3.9	5.6	7.7	9.1	10.5	15.6
**Propadiene**	0.3	0.5	0.7	0.7	0.9	0.9	1.6	1.7	2.3	2.8	2.2
**n-Butane**	tr.	tr.	tr.	tr.	tr.	0.1	tr.	tr.	tr.	tr.	tr.
***(E)*-2-Butene**	0.2	0.2	0.2	0.2	0.2	0.2	0.2	0.2	0.2	0.2	0.2
**1-Butene**	1.5	1.7	1.6	1.6	1.8	1.6	1.9	2.0	2.1	2.1	2.3
**Isobutene**	0.6	0.9	1.6	2.1	2.8	3.3	4.4	6.2	7.3	8.6	12.2
***(Z)*-2-Butene**	0.2	0.1	0.2	0.2	0.2	0.2	0.2	0.2	0.2	0.2	0.2
**Propyne**	0.4	0.5	0.7	0.5	0.5	0.6	0.8	1.0	1.2	1.3	1.4
**Butadiene**	4.2	3.6	3.4	3.5	3.4	3.2	2.9	2.8	2.5	2.3	1.7
**Hexane**	2.3	2.5	2.0	2.3	2.7	2.0	1.8	1.3	1.0	0.8	tr.
**Cyclopentadiene**	0.4	0.2	0.3	0.4	0.4	0.4	0.4	0.5	0.5	0.6	0.6
**Other NA C5–C6**	0.6	0.5	0.6	0.6	0.6	0.6	0.7	0.8	0.8	1.0	1.0
**Benzene**	1.2	0.9	1.0	0.9	0.8	0.9	0.9	1.0	1.1	1.2	1.1
**Toluene**	0.1	0.1	0.1	0.2	0.1	0.2	0.2	0.2	0.3	0.3	tr.
**Ethylbenzene**	tr.	tr.	tr.	tr.	tr.	tr.	tr.	tr.	tr.	tr.	tr.
**m- +p-Xylene**	tr.	tr.	tr.	tr.	tr.	tr.	tr.	tr.	tr.	tr.	tr.
**Styrene+o-Xylene**	tr.	tr.	tr.	tr.	tr.	tr.	tr.	tr.	tr.	tr.	tr.
**Naphthalene**	tr.	tr.	tr.	tr.	tr.	tr.	tr.	tr.	tr.	tr.	tr.
**Other C7–C12**	tr.	tr.	0.1	0.2	0.1	0.2	0.2	0.2	0.3	0.2	tr.
**C12+**	tr.	0.1	tr.	0.1	0.1	0.1	0.1	0.1	0.1	tr.	tr.

Table S9

Co-pyrolysis of isobutane and cyclohexane at 820 °C. Composition of reaction mixture after co-pyrolysis (in columns – content of isobutane in the feedstock)

Isobutane, %	2.2	6.5	10.2	17.3	30.6	38.6	41.7	55.0	70.2	78.5
**Hydrogen**	1.2	1.1	1.1	0.7	0.8	1.2	0.8	1.0	1.0	0.9
**Methane**	3.5	5.3	5.4	8.5	9.5	8.9	11.7	12.2	14.8	15.5
**Ethane**	0.7	0.6	0.6	0.9	1.1	1.0	1.3	1.2	1.5	1.7
**Ethylene**	32.2	37.0	36.0	36.6	33.5	28.7	31.2	24.2	20.3	18.1
**Propane**	0.1	0.1	0.1	0.2	0.3	0.3	0.3	0.4	0.4	0.4
**Propylene**	5.4	6.9	7.2	10.6	12.4	12.2	14.5	16.4	19.5	20.9
**Acethylene**	1.3	1.9	1.8	1.9	1.8	1.3	1.7	1.2	1.2	1.1
**Isobutane**	0.6	1.6	2.0	4.1	5.5	6.7	6.3	9.5	10.9	12.3
**Propadiene**	0.2	0.3	0.4	0.6	0.9	1.0	0.9	1.1	1.5	1.9
**n-Butane**	tr.	tr.	tr.	tr.	tr.	tr.	tr.	tr.	tr.	0.1
***(E)*-2-Butene**	0.2	0.1	0.1	0.1	0.1	0.2	0.2	0.2	0.2	0.2
**1-Butene**	0.3	0.4	0.4	0.7	0.9	0.9	1.0	1.3	1.6	1.8
**Isobutene**	0.3	1.0	1.2	2.7	3.8	4.6	5.0	7.3	8.9	9.8
***(Z)*-2-Butene**	0.1	0.1	0.1	0.1	0.1	0.2	0.1	0.2	0.2	0.2
**Propyne**	0.4	0.6	0.6	0.7	0.8	0.7	1.0	1.1	1.4	1.5
**Butadiene**	22.5	20.7	20.5	16.0	14.3	14.2	11.9	10.1	7.0	5.5
**Cyclohexane**	19.4	11.3	11.4	5.9	5.6	6.5	3.2	3.2	1.6	1.3
**Cyclopentadiene**	1.9	1.1	1.2	0.8	0.7	1.5	0.8	1.3	1.0	0.9
**Other NA C5–C6**	2.2	1.8	1.9	1.6	1.5	2.0	1.6	1.9	1.7	3.0
**Benzene**	5.5	6.1	6.1	5.7	5.0	5.1	4.9	4.2	3.4	1.3
**Toluene**	0.7	0.6	0.6	0.6	0.6	0.9	0.7	0.8	0.7	tr.
**Ethylbenzene**	tr.	tr.	tr.	tr.	tr.	0.1	tr.	0.1	0.1	0.1
**m- +p-Xylene**	0.1	0.1	tr.	tr.	tr.	0.1	tr.	0.1	0.1	0.1
**Styrene+o-Xylene**	0.3	0.3	0.3	0.2	0.1	0.3	0.2	0.3	0.2	0.2
**Naphthalene**	0.1	0.1	0.1	tr.	tr.	0.1	tr.	tr.	tr.	tr.
**Other C7–C12**	0.7	0.7	0.4	0.4	0.4	0.8	0.4	0.6	0.5	1.2
**C12+**	0.2	0.3	0.3	0.3	0.2	0.5	0.2	0.2	0.2	0.2

Table S10

Co-pyrolysis of isobutene and 3-methylpentane at 820 °C. Composition of reaction mixture after co-pyrolysis (in columns – content of isobutene in the feedstock)

Isobutene, %	4.5	17.7	27.0	35.1	41.4	55.3	72.4
**Hydrogen**	0.4	0.4	0.2	0.3	0.3	0.2	tr.
**Methane**	17.2	16.5	16.5	16.9	15.7	13.8	13.3
**Ethane**	4.0	3.3	3.1	2.7	2.4	2.1	1.7
**Ethylene**	37.9	32.2	30.1	27.2	24.0	18.4	12.7
**Propane**	0.4	0.3	0.3	0.2	0.2	0.1	0.1
**Propylene**	17.8	17.8	17.6	16.5	16.5	15.5	13.2
**Acethylene**	0.8	0.9	0.9	1.1	1.0	0.8	0.7
**Isobutane**	tr.	tr.	tr.	tr.	tr.	tr.	tr.
**Propadiene**	0.8	1.2	1.5	1.9	2.3	3.1	3.9
**n-Butane**	tr.	tr.	tr.	tr.	tr.	tr.	tr.
***(E)*-2-Butene**	0.8	0.9	0.8	0.7	0.8	0.8	0.6
**1-Butene**	2.0	2.2	2.0	1.7	2.0	2.1	1.5
**Isobutene**	3.1	6.8	9.3	10.8	14.3	22.9	33.5
***(Z)*-2-Butene**	0.8	0.8	0.8	0.7	0.8	0.8	0.6
**Propyne**	0.8	1.3	1.6	2.2	2.5	2.9	3.6
**Butadiene**	6.8	6.4	5.8	5.6	5.1	3.9	2.7
**3-methylpentane**	1.1	1.4	1.0	0.6	1.0	1.4	0.7
**Cyclopentadiene**	1.3	1.8	1.9	2.0	2.3	2.2	2.0
**Other NA C5–C6**	1.2	1.9	1.9	2.0	2.5	3.0	2.8
**Benzene**	1.8	2.1	2.6	3.9	3.4	3.1	3.3
**Toluene**	0.5	0.7	0.9	1.4	1.3	1.3	1.5
**Ethylbenzene**	tr.	0.1	0.1	0.1	0.1	0.1	0.1
**m- +p-Xylene**	0.1	0.1	0.1	0.1	0.2	0.1	0.2
**Styrene+o-Xylene**	0.1	0.2	0.2	0.3	0.3	0.2	0.2
**Naphthalene**	tr.	tr.	tr.	tr.	tr.	tr.	tr.
**Other C7–C12**	0.3	0.5	0.6	0.7	0.8	0.8	1.0
**C12+**	0.1	0.1	0.1	0.3	0.2	0.2	0.2

Table S11

Co-pyrolysis of 2-butene and 3-methylpentane at 820 °C. Composition of reaction mixture after co-pyrolysis (in columns – content of 2-butene in the feedstock)

2-butene, %	3.4	8.5	15.9	14.0	34.5	48.1	69.3	81.6
**Hydrogen**	0.4	0.4	0.3	0.3	0.1	0.1	0.1	0.2
**Methane**	18.2	18.8	19.1	17.8	17.4	16.3	14.3	12.9
**Ethane**	3.6	3.4	3.1	3.7	3.1	2.7	2.0	1.5
**Ethylene**	37.5	37.5	36.9	36.0	31.9	27.9	20.5	15.8
**Propane**	0.4	0.4	0.4	0.3	0.2	0.2	0.1	0.1
**Propylene**	17.3	16.8	16.4	18.0	19.2	20.0	21.0	20.8
**Acethylene**	0.8	0.9	1.0	1.1	1.0	1.1	1.1	1.1
**Isobutane**	tr.	tr.	tr.	tr.	tr.	tr.	tr.	tr.
**Propadiene**	0.6	0.6	0.6	0.7	0.6	0.6	0.5	0.5
**n-Butane**	tr.	tr.	tr.	tr.	tr.	tr.	tr.	tr.
***(E)*-2-Butene**	0.8	0.7	0.7	1.0	1.4	2.0	3.8	5.5
**1-Butene**	1.9	1.6	1.4	1.8	1.8	2.0	2.5	2.6
**Isobutene**	2.0	1.7	1.6	1.6	1.2	1.0	0.7	0.5
***(Z)*-2-Butene**	0.7	0.6	0.6	0.9	1.2	1.7	3.3	4.9
**Propyne**	0.7	0.8	0.8	0.9	0.8	0.9	0.9	0.9
**Butadiene**	7.7	7.9	8.0	9.0	11.7	14.2	19.0	22.7
**3-methylpentane**	0.9	0.6	0.4	0.7	0.5	0.5	0.5	0.3
**Cyclopentadiene**	1.4	1.4	1.6	1.3	1.4	1.5	1.6	1.4
**Other NA C5–C6**	1.3	1.3	1.4	1.3	1.5	1.8	2.4	2.6
**Benzene**	2.4	3.1	3.8	2.6	3.2	3.6	3.8	3.6
**Toluene**	0.6	0.8	0.9	0.6	0.8	0.8	0.8	0.8
**Ethylbenzene**	tr.	0.1	0.1	tr.	0.1	0.1	0.1	0.1
**m- +p-Xylene**	tr.	0.1	0.1	0.1	0.1	0.1	0.1	0.1
**Styrene+o-Xylene**	tr.	0.2	0.2	0.2	0.2	0.2	0.1	0.2
**Naphthalene**	tr.	tr.	tr.	tr.	tr.	tr.	tr.	tr.
**Other C7–C12**	0.5	0.5	0.4	0.3	0.6	0.4	0.5	0.7
**C12+**	tr.	tr.	0.3	tr.	0.2	0.3	0.3	0.2

Table S12

Co-pyrolysis of isobutene and heptane at 820 °C. Composition of reaction mixture after co-pyrolysis (in columns – content of isobutene in the feedstock)

Isobutene, %	5.0	9.4	17.1	18.5	22.0	25.0	30.6	37.1	58.6	67.8	78.1
**Hydrogen**	0.9	0.8	0.6	0.7	0.8	0.4	0.5	0.4	0.1	0.1	tr.
**Methane**	11.8	11.5	11.8	11.7	9.7	12.8	11.3	12.1	12.9	11.7	13.0
**Ethane**	2.3	2.3	2.2	2.0	1.9	2.0	1.8	1.8	1.6	1.2	1.2
**Ethylene**	59.8	58.2	55.4	54.1	51.5	50.9	46.1	43.5	32.2	24.0	18.1
**Propane**	0.4	0.4	0.3	0.3	0.4	0.3	0.3	0.2	0.1	0.1	0.1
**Propylene**	12.8	12.9	13.1	13.2	13.5	13.3	13.5	13.3	12.0	11.0	9.7
**Acethylene**	0.7	0.8	0.8	0.8	0.9	0.7	0.7	0.7	0.8	0.7	0.6
**Isobutane**	0.4	0.5	0.7	0.7	1.0	0.9	tr.	tr.	tr.	tr.	tr.
**Propadiene**	tr.	tr.	tr.	tr.	tr.	tr.	1.3	1.4	2.5	3.2	3.6
**n-Butane**	tr.	tr.	tr.	tr.	tr.	tr.	tr.	tr.	tr.	tr.	tr.
***(E)*-2-Butene**	0.2	0.2	0.2	0.2	0.2	0.2	0.2	0.2	0.2	0.2	0.3
**1-Butene**	1.2	1.3	1.5	1.4	2.4	1.5	2.0	1.7	1.4	1.5	1.1
**Isobutene**	1.1	2.1	3.8	4.4	6.0	6.8	9.7	11.6	21.1	29.5	37.4
***(Z)*-2-Butene**	0.2	0.2	0.2	0.2	0.2	0.2	0.2	0.2	0.3	0.3	0.4
**Propyne**	0.4	0.5	0.6	0.7	0.8	0.8	1.1	1.3	2.3	2.8	3.0
**Butadiene**	3.8	3.7	3.5	3.7	3.6	3.2	3.2	3.1	2.4	1.9	1.5
**Heptane**	1.6	1.7	2.2	1.7	3.6	2.0	3.1	2.4	1.6	2.1	1.3
**Cyclopentadiene**	0.3	0.4	0.5	0.6	0.5	0.7	0.9	1.1	1.3	1.5	1.3
**Other NA C5–C6**	0.4	0.5	0.6	0.7	0.9	0.8	1.2	1.3	1.5	2.3	1.9
**Benzene**	1.2	1.4	1.4	1.7	1.2	1.7	1.6	2.2	3.3	3.2	3.2
**Toluene**	0.2	0.3	0.3	0.4	0.3	0.4	0.4	0.6	1.1	1.3	1.2
**Ethylbenzene**	tr.	tr.	tr.	tr.	tr.	tr.	tr.	0.1	0.1	0.1	0.1
**m- +p-Xylene**	tr.	tr.	tr.	tr.	tr.	tr.	tr.	0.1	0.1	0.1	0.1
**Styrene+o-Xylene**	tr.	0.1	tr.	0.1	tr.	0.1	0.1	0.1	0.2	0.2	0.1
**Naphthalene**	tr.	tr.	tr.	tr.	tr.	tr.	tr.	tr.	tr.	tr.	tr.
**Other C7–C12**	0.3	0.3	0.3	0.4	0.5	0.3	0.4	0.5	0.6	0.9	0.6
**C12+**	0.1	0.1	tr.	0.2	0.1	0.1	0.1	0.1	0.2	0.2	0.2
